# Complement Protein C3 Suppresses Axon Growth and Promotes Neuron Loss

**DOI:** 10.1038/s41598-017-11410-x

**Published:** 2017-10-10

**Authors:** Sheri L. Peterson, Hal X. Nguyen, Oscar A. Mendez, Aileen J. Anderson

**Affiliations:** 1 Sue & Bill Gross Stem Cell Center, University of California, Irvine, Irvine, CA 92697 USA; 2 Institute for Memory Impairments and Neurological Disorders, University of California, Irvine, Irvine, CA 92697 USA; 3 Department of Anatomy & Neurobiology, University of California, Irvine, Irvine, CA 92697 USA; 4 Department of Physical Medicine and Rehabilitation, University of California, Irvine, Irvine, CA 92697 USA

## Abstract

The inflammatory response to spinal cord injury (SCI) involves localization and activation of innate and adaptive immune cells and proteins, including the complement cascade. Complement C3 is important for the classical, alternative, and lectin pathways of complement activation, and its cleavage products C3a and C3b mediate several functions in the context of inflammation, but little is known about the potential functions of C3 on regeneration and survival of injured neurons after SCI. We report that 6 weeks after dorsal hemisection with peripheral conditioning lesion, C3^−/−^ mice demonstrated a 2-fold increase in sensory axon regeneration in the spinal cord in comparison to wildtype C3^+/+^ mice. *In vitro*, addition of C3 tripled both myelin-mediated neurite outgrowth inhibition and neuron loss versus myelin alone, and ELISA experiments revealed that myelin serine proteases cleave C3 to generate active fragments. Addition of purified C3 cleavage products to cultured neurons suggested that C3b is responsible for the growth inhibitory and neurotoxic or anti-adhesion activities of C3. These data indicate that C3 reduces neurite outgrowth and neuronal viability *in vitro* and restricts axon regeneration *in vivo*, and demonstrate a novel, non-traditional role for this inflammatory protein in the central nervous system.

## Introduction

Multiple factors contribute to central nervous system (CNS) axon regeneration failure. These include: neuronal cell death due to primary and secondary injury, the reduced intrinsic growth state of adult central neurons^1^, the physical barrier introduced by glial scar formation^2^, the growth inhibitory proteins secreted by scar astrocytes such as chondroitin sulfate proteoglycans^3–5^, the growth inhibitory proteins in disrupted and intact myelin (e.g., Nogo, Oligodendrocyte-Myelin glycoprotein, and Myelin-Associated Glycoprotein)^6–9^, and the up-regulation of developmental guidance cues such as semaphorin, slit, and ephrin^10^. A number of pathways and molecules modulating these factors have been identified, for example, cAMP and mTOR/PTEN signaling^11,12^. In parallel, we have recently reported a novel role for complement C1q in this capacity, demonstrating C1q binding to MAG within myelin, and modulation of axon growth and guidance by C1q in culture and *in vivo* following spinal cord injury (SCI)^13^.

Complement is an auto-catalytic cascade of inflammatory proteins with well described immunologic functions in inflammatory cell recruitment and activation, opsonization of pathogens and cellular debris for phagocytic removal, and direct cell lysis. Many complement proteins and inhibitors, including C3, C1q, Factor B, Factor H, C4, and membrane attack complex (MAC), are increased in the spinal cord after SCI or spinal root injury in rodents and humans^14–21^. These proteins are present in the spinal cord in association with axons and myelin over a wide range of time-points and tissue locations, although the roles for complement proteins in this context are not well characterized^22^. One putative function of complement protein-myelin interactions is to facilitate myelin phagocytosis^23,24^, and association of both C3 fragments and C1q protein with myelin has been reported *in vitro* and *in vivo* ^25–27^. Further, complement proteins may perform non-immune, or “non-traditional,” functions in CNS development and plasticity, including tissue regrowth, cell migration, proliferation, differentiation, survival, and synaptic remodeling^22^.

Complement C3 is a 185 kDa protein that serves as the point of convergence for immune activation of the three major complement pathways: classical, alternative, and lectin. *In vivo*, the immune functions of complement C3 are mediated by C3 cleavage products, C3a and C3b, as well as fragments produced by additional processing of these products. In this report, we investigate the potential role for complement C3 in the modulation of axon regeneration and neuronal survival after SCI using both *in vivo* and *in vitro* models.

## Materials and Methods

All experiments were carried out under a protocol (#2002–2259) approved by the Institutional Animal Care and Use Committee (IACUC) at University of California, Irvine, according to the guidelines of the IACUC and consistent with federal guidelines for the care and use of laboratory animals. All groups and treatments were randomized, and all surgeries, exclusions, tissue processing, imaging, and analyses were performed blinded to culture condition or mouse genotype, and all groups/tissues tested for comparison were processed in parallel. See Table 1 for summary of groups and methods for *in vivo* experiments.

**Table 1:** Summary of Groups and Methods for *In Vivo* Axon Regeneration and Neuronal Survival Experiments.

Injury Group Name	T8 dorsal hemisection spinal cord injury (day 0)	Sciatic nerve transection (day 0)	Sciatic nerve transection (day 7)	Post-injury survival time (days)	Tracer Injection	Genotypes Tested	Figures
Dorsal hemisection SCI with sciatic nerve conditioning injury	Yes	Yes	Yes	42	CTB	C3^+/+^ & C3^−/−^	Fig. 1
Dorsal hemisection SCI (“SCI Only”)	Yes	Sham	Sham	42	CTB	C3^+/+^	Fig. 1
Sciatic nerve transection	No	Yes	No	10	No	C3^+/+^ & C3^−/−^	Fig. 2
Uninjured	No	No	No	10	No	C3^+/+^ & C3^−/−^	Fig. 2

Injury group names listed here are used throughout the text, alongside genotype, to describe each experimental group.

## Dorsal hemisection SCI with sciatic nerve conditioning injury

Wildtype C3^+/+^ mice used in this study were litter-mates of C3^−/−^ mice. See Supplemental Information for additional mouse details. *Mouse surgeries*: All surgical procedures were performed using sterile technique with mice under isofluorane anesthesia. Dorsal hemisection was performed on 25 male mice, N = 9 C3^−/−^ and N = 16 C3^+/+^, 5–8 months old. The spinal cord was exposed at thoracic level ~T8 by dorsal laminectomy, followed by bilateral dorsal column transection using marked micro-dissection scissors (spinal level was confirmed for all mice as T6, T7, T8, or T9 by spinal root at time of tissue harvest). N = 9 C3^−/−^ and N = 10 C3^+/+^ mice received sciatic nerve injury immediately after SCI in a conditioning lesion paradigm. Blunt dissection to the left sciatic nerve at mid-thigh level was followed by ligature placement and sciatic nerve transection distal to the ligature. The remaining N = 6 C3^+/+^ mice received sham sciatic nerve surgeries (SCI only), in which blunt dissection was performed without sciatic nerve cut; these mice served as a validation control for the conditioning lesion paradigm. After suturing, mice were monitored and maintained on water-jacketed heating pads at 37 °C while recovering from anesthesia. A second surgery was conducted 7 d after the first, during which mice received either a second left sciatic nerve transection proximal to the first transection^28^, or a second sham sciatic nerve surgery. Mice were maintained on acidified water and antibiotics, and treated for pain as necessary. Bladder expression was performed 2–3x/day by manual crede. Five days prior to tissue collection, the left sciatic nerve was exposed again and 1.5 μl of 10 mg/mL cholera toxin β subunit (CTB, List Biological Laboratories), a trans-ganglionic tracer, was injected into the proximal nerve section using a pulled glass capillary tube pipette. *Tissue Collection*: Mice were sacrificed 6 weeks after the initial surgery by lethal dose of sodium pentobarbital and cardiac perfusion of saline followed by 4% paraformaldehyde. Spinal cord segments T4-T9 (identified by spinal root) and L1-L6 (identified by vertebral count), as well as brain and left sciatic nerve were harvested, sunk in 4% paraformaldehyde/10% sucrose, and flash-frozen with isopentane. A sliding microtome was used to section frozen thoracic spinal cord (50 μm horizontal), lumbar spinal cord (50 μm horizontal) and brain (30 μm coronal) from each mouse.

### Axon regeneration immunohistochemistry and analyses

For analyses of regenerated axons, T4-T9 spinal cord segments were processed for CTB immunoreactivity, and length of the longest CTB^+^ axon was determined by visually scanning each spinal cord section, and measuring rostro-caudal length from the center of the injury to the most rostral axonal tracer label in each animal. The center of the injury was set to 0 μm, with rostral positions reported as positive and caudal as negative. Lesion volume was determined from sections containing visible injury using the unbiased, systematic random sampling methods of stereology (Cavalieri probe). Exclusions were made based on insufficient tracer labeling (lumbar), incomplete dorsal column lesions (brain), and early death or euthanasia. Final animal Ns for histological analyses were: C3^+/+^ N = 5; C3^−/−^ N = 5; C3^+/+^ SCI only N = 2. See Supplemental Information for additional immunohistochemistry, analysis, and exclusion details.

### Sciatic nerve transection, immunohistochemistry, and analysis of DRG survival

Five adult male C3^−/−^ mice and 3 age-matched male C3^+/+^ mice underwent a single transection of the left sciatic nerve and were perfused with paraformaldehyde 10 days post-injury using the procedures described above. Lumbar L4 and L5 dorsal root ganglia (DRGs) were harvested from both the left and right side of each mouse (by spinal root count), sunk in para-sucrose, and flash frozen as described above. A cryostat with a tape-transfer system (CryoJane) was used to section (30 μm) all DRGs (4/mouse).

For estimates of total DRG neuron number, a series of every 4th section of each DRG (4–11 sections/RG, 90 μm apart) was processed for NF200 immunoreactivity as detailed in Supplemental Information. Histological sections were examined on a Zeiss Axio Imager M2 microscope at 400× magnification. Estimated L4 + L5 DRG neuron counts were determined using unbiased, systematic random sampling by stereology with the optical fractionator probe (MicroBrightField v11.01 Software). Using a 150 μm × 150 μm grid and a 90 μm × 90 μm counting frame, Gunderson CE m = 1 ranged 0.05–0.09 for all DRGs.

### Dissociated primary cortical cell cultures for neurite outgrowth

Myelin substrate (isolation of myelin substrate is described in Supplemental Information) was plated at 7.5 μg/mL (1.12 μg/well) on pre-coated poly-L-lysine (0.005% PLL; Sigma) 8-well permanox chamber slides (Lab-Tek) and adsorbed by overnight desiccation under vacuum. Complement C3 protein was then added to appropriate wells (Quidel, 250 μg/ml, 95 μl, 680 nM). After 2 h incubation, wells were washed once to remove unbound protein prior to plating dissociated neonatal (P0-P4) Sprague Dawley rat cortical cells. The dissected cerebral cortex was minced and resuspended in NB media (Invitrogen) containing papain (Worthington, 2 mg/mL), and incubated with agitation for 20 m. Cell solution was triturated in NB media containing 10% FBS, then filtered, pelleted, and resuspended in NB media. An Optiprep (Sigma) and MOPS buffer solution was diluted with NB media to form a density gradient for separation of neurons from myelin, cell debris, microglia, and RBCs, as described previously^29^. The neuronal layer was isolated and diluted (50,000 cells/mL, 0.2 mL/well) with NB media containing B-27 supplement (Invitrogen), glutamax (Invitrogen), and penicillin-streptomycin (Invitrogen). Cells were plated in each well of control, myelin only, or myelin with C3. These experiments also included a myelin with C1q treatment group, as reported previously^13^. Cells were incubated for 48 h before fixation with 2% paraformaldehyde, followed by immunocytochemistry and microscopic analysis. Cortical cultures were chosen (instead of DRG cultures) for these experiments because we reliably observe myelin inhibition in cortical cultures, and to enable comparison with our previous report for complement C1q^13^.

### C3a ELISA (enzyme-linked immunosorbent assay)

C3a concentration in solution containing either Hepes control, myelin alone, C3 alone (Quidel, 67 μg/mL), myelin with C3 (Quidel, 67 μg/mL), or myelin with C3 (Quidel, 67 μg/mL) and FUT175, after 1 day incubation at 37 °C, was measured using the MicroVue C3a Enzyme Immunoassay Kit (Quidel) according to manufacturer’s directions. Two independent replicates were performed, each with duplicate wells.

### Neurite outgrowth assay in dissociated primary DRG cell cultures

Dissociated adult mouse DRG cells were plated with various concentrations of purified complement protein C3, C3a, C3b, or C3a desArg onto pre-coated poly-L-lysine (0.005% PLL; Sigma) 8-well permanox chamber slides. First, adult (C57BL6/J) mice were killed and DRGs from both sides of lumbar and thoracic vertebrae were removed and treated with 0.5 mg/mL trypsin (Sigma) and 1 mg/mL collagenase (Sigma) for 20 min at 37°C. Cells were gently triturated in NB-A media (Invitrogen) with 10% FBS (Gibco) and filtered through a cell strainer, then pelleted and resuspended in NB-A with 2% FBS and complement proteins to 10,000 cells/mL (0.2 mL/well). Immediately following cell plating, complement proteins C3, C3a, C3b, and C3a desArg (CompTech), were added to media at 0.01 μg/ml–1 μg/ml (specifically: 0.01 μg/ml (54 pM), 0.10 μg/ml (540 pM), and 1.00 μg/ml (5.4 nM) C3; 0.01 μg/ml (57 pM), 0.10 μg/ml (570 pM), and 1.00 μg/ml (5.7 nM) C3b; 0.01 μg/ml (1 nM), 0.10 μg/ml (56 nM), and 1.00 μg/ml (111 nM) C3a; 0.01 μg/ml (1 nM), 0.05 μg/ml (5.6 nM), and 0.10 μg/ml (11 nM) C3a desArg). Each experiment tested a concentration curve for a particular protein (starting at 0 μg/ml), with a single protein tested per biological replicate (3–4 wells in each of 3–4 biological replicates, approximately 1000 neurons/condition). The parameters for complement concentration, DRG plating density, and time in culture were determined in a series of pilot experiments. After 48–72 h, cells were fixed with 2% paraformaldehyde, followed by immunocytochemistry and microscopic analysis.

### Immunocytochemistry and quantification for *in vitro* neurite analyses

Fixed cell cultures were immunolabeled for β-tubulin class III with Hoechst counterstain, as detailed in Supplemental Information. Images were captured, β-tubulinIII+ and Hoechst+ cells quantified (by hand or using a custom protocol in Bitplane Imaris), and the following neurite analyses were performed: *Longest neurite length per neuron*, *Neurite count per neuron*, *Total neurite length per well*, and *Average neurite length per neuron* (by ImageJ or using a custom protocol in Bitplane Imaris), as detailed in Supplemental Information. For cortical and DRG neuron cultures, 3–4 independent experiments (each with 2–4 wells per condition) were combined for figures and statistics, therefore each culture condition represents data collected from approximately 500–1500 neurons. To integrate independent biological replicates into the same plot and perform statistics, data is represented for each treatment as the percent of the control well result for that measure in the same biological replicate (% Control = mean of treatment wells/mean of control wells * 100).

### Statistical analysis

See individual methods sections for details on number of data points, wells, independent replicates, and mice for each experiment. Statistical comparison of C3^−/−^ versus C3^+/+^ tissue for each histological endpoint was performed using Student’s t-test (2-tailed for regeneration and lesion volume analyses and 1-tailed for DRG neuron count in WT controls) or one-way ANOVA (DRG neuron count). Correlation between axon length and lesion volume was tested by linear regression analysis. For the myelin + C3 cortical neurite outgrowth experiments, a Student’s t-test (2-tailed) comparing myelin and myelin + C3 treatment was used. For the C3a ELISA, ANOVA analysis followed by Dunnett’s post-test was used to compare each group versus C3 alone. For the C3/C3a/C3b/C3a desArg morphological assays, ANOVA analysis followed by Dunnett’s post-test was used to compare the normalized treatment data to the associated control data for each output measure. Outliers in technical replicates for culture experiments were detected using Grubb’s outlier test, and outlier wells were removed from analysis. Statistical significance was defined as *P < 0.05, **p < 0.01.

## Results

### Complement C3^−/−^ mice display increased sensory axon regeneration in the spinal cord after dorsal hemisection SCI with sciatic nerve conditioning injury

**Figure 1:**
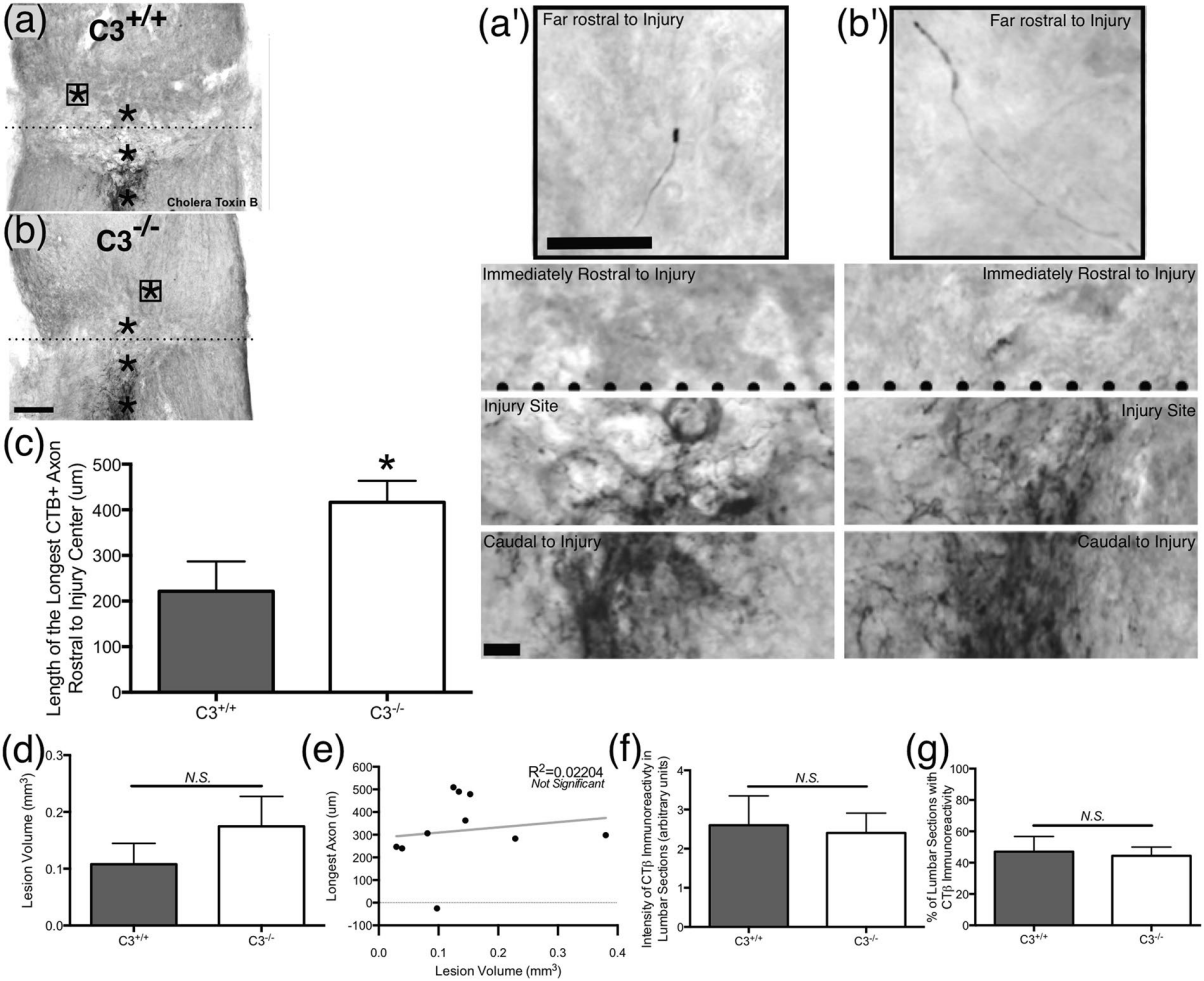
Complement C3 knockout mice displayed increased sensory axon regeneration in the spinal cord following dorsal hemisection SCI with sciatic nerve conditioning injury. Horizontal spinal cord sections from a (**a**) C3^+/+^ mouse and a (**b**) C3^−/−^ mouse 6 weeks after dorsal hemisection SCI with conditioning injury with CTB tracer in black, the injury epicenter marked with a dotted line, and asterisks (*) denoting the areas enlarged in the corresponding panels on the right (**a′,b′**); boxed (□) asterisk (*) indicates the furthest (most rostral) regenerating axon in the section. (**c**) Traced, severed, regenerating sensory axons grew significantly longer (more rostral) in C3^−/−^ mice than in C3^+/+^ littermates. (**d**) There was no difference in lesion volume between genotypes. (**e**) Regenerated axon length was not correlated to lesion volume (R^2^ = 0.022). Importantly, CTB tracer immunoreactivity in the lumbar spinal cord was similar between genotypes, as assessed by (**f**) staining intensity score and (**g**) percent of total sections with positive label. N = 5 C3^−/−^, N = 5 C3^+/+^. Mean ± SEM. *p < 0.05 Student’s t-test (2-tailed). Scale bar (**a**,**b**) = 500 μm; (**a′**,**b′**) = 70 μm & 120 μm.

To evaluate the potential for both growth promoting and growth inhibitory effects of C3 on axon regeneration in our *in vivo* studies, we employed a peripheral conditioning SCI model, which involves injury of the dorsal spinal cord as well as the sciatic nerve, and is known to induce ascending axon regeneration. The gracile fasciculus of the spinal cord is an ascending sensory tract composed of the central axon branches of DRG neurons, while the sciatic nerve contains the peripheral axon branches of lumbar DRG neurons. The dorsal hemisection SCI with sciatic nerve conditioning injury model produces modest regeneration of gracile fasciculus axons after injury of both the central (T8 dorsal column transection on day 0) and peripheral (sciatic nerve transection on days 0 and 7) DRG branches, in contrast to the exclusive axon die-back that results from central injury alone^28^. Accordingly, we assessed regeneration of gracile fasciculus axons in C3^+/+^ and C3^−/−^ mice 6 weeks post-dorsal hemisection SCI with sciatic nerve conditioning injury, using CTB tracer injected into sciatic nerve to identify centrally transected axons of peripherally conditioned neurons (Fig. 1a,b). We validated that the peripheral conditioning SCI model produced expected results in C3^+/+^ mice by including two C3^+/+^ mice that had SCI but no conditioning injury. These mice exhibited maximum axon lengths of 170 μm and 39 μm caudal (−) to the lesion (data not shown), while C3^+/+^ mice that had SCI plus conditioning demonstrated axons regenerating through the lesion and rostral (+) to it (Fig. 1c; +222 ± 65 μm).

On average, we found the longest (most rostral) traced axon in C3^−/−^ mice to be nearly double that of C3^+/+^ mice (Fig. 1c; 416 ± 47 vs. 222 ± 65 μm, *p = 0.042). Importantly, lesion volume did not differ between groups (Fig. 1d; 0.108 ± 0.037 vs. 0.175 ± 0.053 mm^3^, p = 0.328) and no correlation between axon length and lesion volume was detected (Fig. 1e; linear regression, R^2^ = 0.022 goodness of fit with slope not different from 0, p = 0.682). Lesion completeness and presence of tracer in tissue were verified for each mouse included in these analyses (see *Materials and methods* section for exclusion criteria and data). While these results support our hypothesis, an increase in CTB uptake or transport could also account for the increased axon length in C3^−/−^ mice. Accordingly, we evaluated the CTB label in the lumbar spinal cord of each mouse in terms of overall intensity (Fig. 1f) and proportion of sections with positive labeling (Fig. 1g), and observed no differences between genotypes (2.6 ± 0.7 vs. 2.4 ± 0.5, p = 0.831; 47 ± 10 vs. 44 ± 6, p = 0.822). The lesion completeness criteria, lesion size comparison, and tracer intensity comparison together suggest that differences in spared axons, lesion size, or tracer abundance did not influence our observations of the effect of C3 knockout on regeneration in this model. Overall, these data indicate that C3 negatively regulates sensory axon regeneration in the spinal cord after SCI.

### Lack of DRG neuron loss following sciatic nerve injury in C3^−/−^ mice

**Figure 2:**
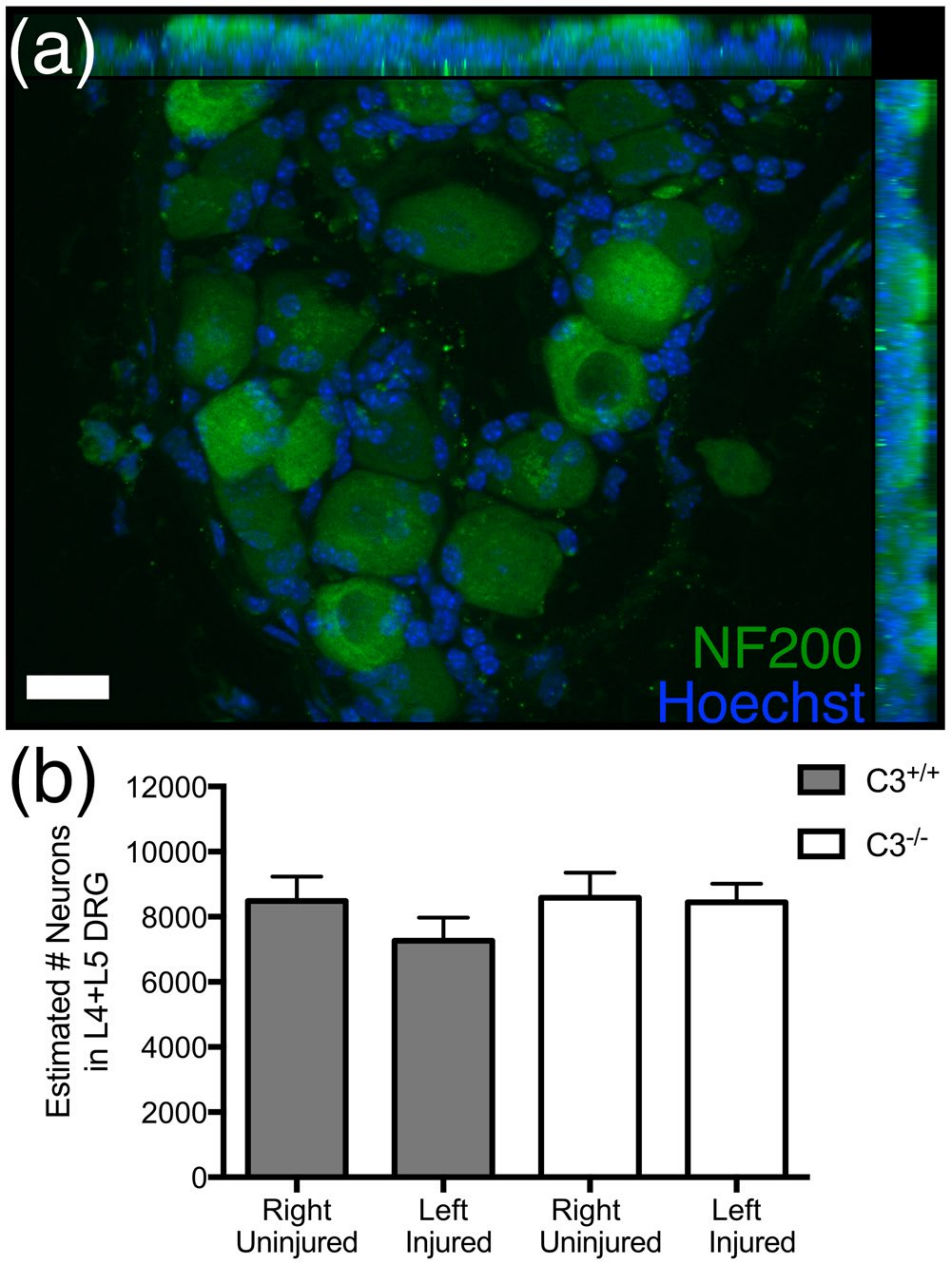
Complement C3 knockout mice did not exhibit DRG neuron loss following sciatic nerve transection. (**a**) Representative section of a right (uninjured) L4 DRG from a C3^+/+^ mouse for quantification, showing immunofluorescent labeling for NF200^+^ DRG neurons (green) with Hoechst counterstain (blue). (**b**) Quantification of L4 + L5 DRG neuron number for uninjured (right) DRGs and injured (left) DRGs in C3^+/+^ and C3^−/−^ mice by stereology demonstrated a lack of injury-induced neuron loss in C3^−/−^ mice (p > 0.05 one-way ANOVA). N = 5 C3^−/−^, N = 3 C3^+/+^. Mean ± SEM. Scale bar = 10 μm.

Injuries that affect the peripheral branch of DRG axons have been reported in some studies to modestly decrease DRG neuron number (6–25% reduction observed at 1–2 weeks post-injury, with a median effect size of ~21%), and increase DRG expression of cell death markers^30–34^. Accordingly, genotype differences in DRG neuronal survival after sciatic nerve injury may potentially underly the observed C3^−/−^ regeneration phenotype. To test this hypothesis, we compared the effect of C3 deficiency on DRG neuron loss following sciatic nerve injury by quantifying neuron number in right versus left L4 and L5 DRGs in C3^+/+^ and C3^−/−^ mice 10 days after left sciatic nerve transection. For these experiments, sciatic nerve injury was not accompanied by dorsal column SCI. To estimate DRG neuron number, immunofluorescent images of Neurofilament-200 labeled DRG sections (Fig. 2a) were quantified using the Stereoinvestigator optical fractionator probe (stereology).

No differences in total estimated L4 + L5 DRG neuron number were detected between any of the groups in one-way ANOVA comparison (Fig. 2b; p = 0.626), or between left (injured) and right (uninjured) DRGs in either the C3^−/−^ (8441 ± 577 vs. 8582 ± 776) or C3^+/+^ (7267 ± 710 vs. 8481 ± 752) mice. A non-significant trend (p = 0.148 one-tailed paired t-test) for reduced neuron number was observed in DRGs from the injured side of C3^+/+^ mice, but was completely absent in the C3^−/−^ mice. While these data cannot exclude the possibility that C3 inhibition or deficiency could be neuroprotective, they suggest that the potential contribution of DRG neuronal loss to DRG regenerative events in BUB/BnJ mice is a small one.

### Complement C3 exacerbates neurite outgrowth inhibition on myelin

**Figure 3:**
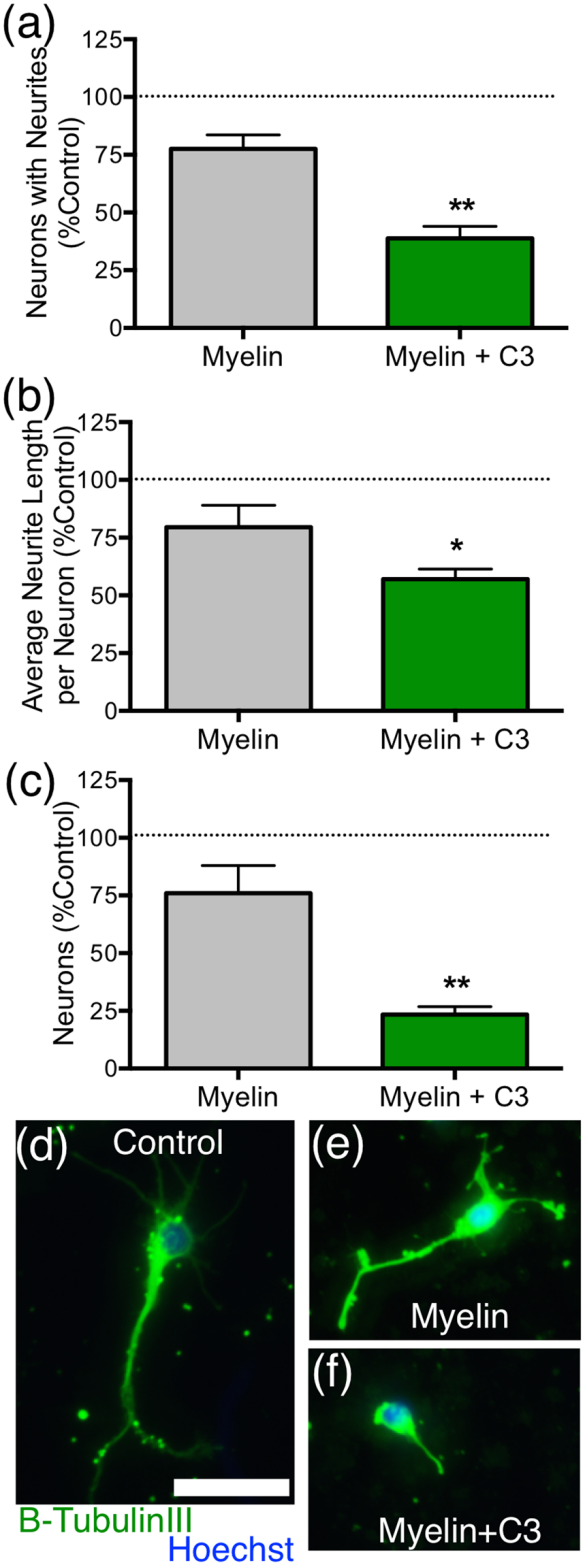
Complement C3 exacerbated myelin-mediated neurite outgrowth inhibition for cultured cortical neurons. (**a**) Percentage of neurons with neurites, (**b**) average neurite length per neuron, and (**c**) number of neurons were each graphed as a percent of the values for untreated wells (control, marked by dotted line) from the same experiment (% control). Myelin plus C3 was significantly more inhibitory to neurite growth and neuron number than myelin alone. Note that neurite growth and number in myelin treated wells is <100% of untreated controls, as expected. Immunofluorescent images of cultured cortical neurons grown in either (**d**) control, (**e**) myelin, or (**f**) myelin +C3 conditions. Immunofluorescent labeling for β-TubulinIII^+^ neurons (green) and Hoescht nuclear counterstain (blue). Scale bars (**d**,**e**,**f**) = 30 μm. Mean ± SEM. *p < 0.05, **p < 0.01 Student’s t-test (2-tailed) vs. myelin alone. N = 4 (myelin); N = 3 (myelin + C3) independent biological replicates.

To further investigate the potential role for C3 in axon regeneration, as well as the underlying mechanism, we next employed a series of *in vitro* models. Given our recent identification of a novel modulatory role for complement C1q in myelin-mediated inhibition of axonal regeneration^13^, the established interaction of C3 and its active products with axons and myelin in the injured CNS, and the observed enhancement in axonal growth in C3^−/−^ mice in the peripheral conditioning model reported here, we hypothesized that C3 would affect primary neurite outgrowth on myelin substrate in culture (Fig. 3). The influence of C3 on neurite growth and neuron survival was evaluated using an assay in which myelin (1.12 μg/well) was adsorbed onto culture wells, followed by addition of C3 (25 μg/well, 680 nM), incubation, and washing before plating of primary dissociated early postnatal rat cortical neurons (Fig. 3). Culture wells with either no treatment or myelin only were included as controls. As reported previously^13^, these cortical cultures contain less than 0.5% Iba-1^+^ macrophages/microglia and do not exhibit C5b-9 assembly, therefore, the contribution of immune cell-derived factors or lytic complement is unlikely to underlie the effects of exogenous C3 administration in this paradigm.

As expected, neurons grown on a myelin substrate for 2 days demonstrated reductions in percentage of *neurons with neurites* (Fig. 3a, 80.6 ± 3.0%) and *average neurite length* per neuron (Fig. 3b, 79.5 ± 9.5%) in comparison to neurons grown in untreated control conditions, which were normalized to 100%. In agreement with our in vivo results, *in vitro* addition of C3 exacerbated neurite outgrowth inhibition on myelin in multiple measures, including percentage of *neurons with neurites* (Fig. 3a; 38.8 ± 5.2%; **p = 0.009 vs. myelin) and *average neurite length* per neuron (Fig. 3b; 57.0 ± 4.4%; *p = 0.044 vs. myelin). Additionally, while the role for C3 in DRG neuron toxicity in vivo following sciatic nerve transection was unclear (Fig. 2), C3 treatment markedly decreased the number of adherent cortical neurons in this assay (Fig. 3c; 23.3 ± 3.5%; **p < 0.007 vs. myelin 76.0 ± 12.0%). Interestingly, these data are in contrast to the rescue effect of C1q in the same model^13^, supporting the specificity of the observed effects, and suggesting a novel and distinct role for C3 as a modulator of myelin-mediated inhibition of axonal growth and neuronal cell loss.

### Myelin cleaves complement C3 via a serine protease mediated mechanism *in vitro*

**Figure 4:**
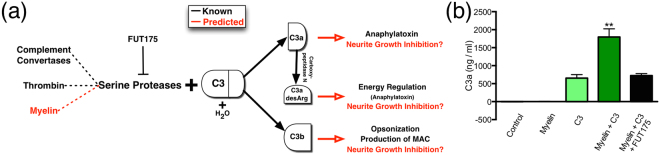
Complement C3 can be cleaved by a serine protease in myelin. (**a**) Diagram of C3 cleavage including tested hypotheses. Complement convertases are known serine proteases that cleave C3 into C3a and C3b, which are functionally active immune proteins; in turn, C3a is converted to C3a desArg by carboxypeptidase N. FUT-175 inhibits serine protease activity and prevents C3 cleavage. Spontaneous hydrolysis of C3 also occurs at a relatively low level generating C3bH_2_0. We predicted (in red) that serine proteases in myelin could cleave C3, and that newly formed active cleavage products (C3b, C3a, C3a desArg) may mediate neurite outgrowth inhibition and/or neuron death. These hypotheses are tested in Figs 4b and 5. (**b**) Results of C3a ELISA following incubation of C3 alone, C3+ myelin, and C3+ myelin + FUT-175. As expected, essentially no C3a was detected in control or myelin wells, and a small amount of C3a was detected with C3 alone. However, myelin significantly increased the generation of C3a, and this effect was blocked by FUT-175, indicating that serine proteases in myelin are capable of C3 cleavage. Mean ± SEM. ***p < 0.001 ANOVA, with **p < 0.01 Dunnett’s post-hoc t-test vs. C3 alone. N = 2 biological replicates for per group.

Because the known immunologic functions for complement C3 are mediated through its principal cleavage products, C3a and C3b, these results suggest that C3 is either cleaved *in vitro* in this paradigm, or that intact C3 is able to exert a biological effect as a stable protein. The conventional mechanism of C3 cleavage is via the serine protease activity of the classical and alternative pathway C3 convertases (C4bC2b and C3bBb). Importantly, C3bBb formation can be triggered spontaneously, via generation of C3(H20) in the fluid phase, enabling Factor B binding and cleavage by Factor D to generate C3(H_2_0)Bb in a process known as tickover (Fig. 4a)^35,36^. Although incubation of whole serum with central or peripheral myelin has been shown to activate the classical and alternative complement pathways, respectively, our *in vitro* experiments involved a purified myelin preparation in the absence of serum, and there is no evidence for the presence of other complement components in these central or peripheral myelin preparations^37^. An alternative mechanism for C3 cleavage involves direct activity by cationic and neutral proteases, including neutrophil elastase, which have been shown to generate active C3a and other derivatives^38–40^. In parallel, non-complement serine proteases, such as those of the coagulation cascade, have also been shown to substitute for complement convertases^41,42^. Together, these data demonstrate that C3 cleavage products can be generated directly, without convertase formation. Accordingly, we hypothesized that myelin-associated serine proteases (e.g. neurosin)^43,44^ could cleave C3 into immunologically active products in our culture paradigm.

We assessed C3 cleavage in the presence and absence of purified myelin proteins in our *in vitro* culture conditions using C3a ELISA (Fig. 4b). C3a was essentially undetectable in untreated control (Fig. 4b; −2 ± 2 ng/ml) and myelin only (1 ± 8 ng/ml) samples. Consistent with the expected C3 hydrolysis, a small amount of C3a was produced with the addition of C3 alone (655 ± 97 ng/ml). Surprisingly, C3a generation was increased approximately 3-fold above spontaneous hydrolysis levels in the presence of both myelin and C3 (1796 ± 227 ng/ml, **p < 0.01 vs. C3 alone). Furthermore, this increase was blocked by addition of the serine protease inhibitor FUT-175 (725 ± 55 ng/ml, p > 0.05 vs. C3 alone). These data indicate that myelin-associated serine proteases are capable of C3 cleavage, suggesting a novel mechanism for C3 fragment generation, and that C3b, C3a, or C3a desArg alone could be sufficient to produce neurite growth inhibition.

### C3b alone is sufficient to induce neurite outgrowth inhibition and reduce neuron number in culture

**Figure 5:**
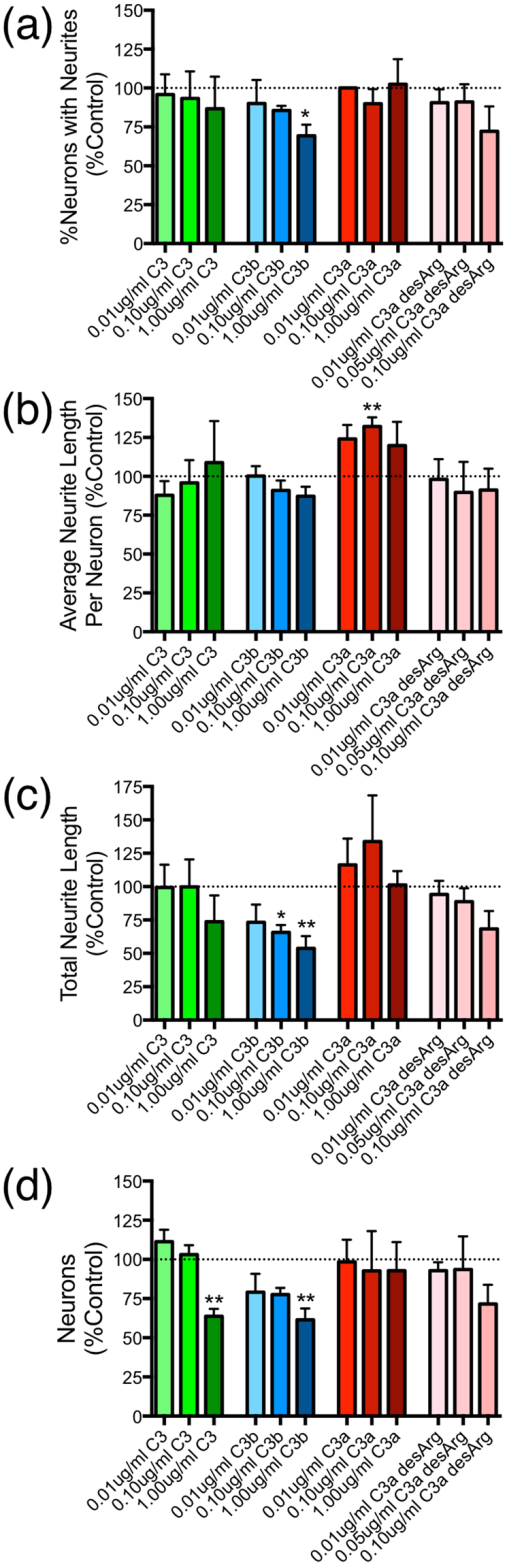
In the absence of myelin, complement C3b was growth inhibitory and promoted neuron loss in DRG cultures. Complement C3, C3a, or C3a desArg alone did not inhibit neurite growth. Quantification of (**a**) percent of neurite-growing neurons, (**b**) average neurite length per neuron, (**c**) total neurite length per image, and (**d**) number of β-TubulinIII^+^ neurons are reported for each treatment: C3 (green bars), C3b (blue bars), C3a (red bars), and C3a desArg (pink bars) at 3 concentrations. Tested concentrations were: 0.01 μg/ml (54 pM), 0.10 μg/ml (540 pM), and 1.00 μg/ml (5.4 nM) C3; 0.01 μg/ml (57 pM), 0.10 μg/ml (570 pM), and 1.00 μg/ml (5.7 nM) C3b; 0.01 μg/ml (1 nM), 0.10 μg/ml (56 nM), and 1.00 μg/ml (111 nM) C3a; 0.01 μg/ml (1 nM), 0.05 μg/ml (5.6 nM), and 0.10 μg/ml (11 nM) C3a desArg. Data is expressed as percent of the control wells from the same experiment, with Mean ± SEM. ANOVA with Dunnett’s post-test vs. control (dashed line), *p < 0.05, **p < 0.01. N = 3–4 independent biological replicates per group.

To test the hypothesis that individual C3 cleavage products are sufficient for neurite outgrowth inhibition *in vitro*, primary dissociated adult mouse DRG neurons were cultured for 2 days in media containing either vehicle control, or C3, C3b, C3a, or C3a desArg (the inactivation product of C3a). No myelin or pre-conditioning was used in these experiments, in order to test the sufficiency of C3 or C3 cleavage products as axonal growth inhibitors. Endogenous production of immune molecules from inflammatory cells is unlikely to confound this exogenous treatment paradigm, because these DRG cultures do not contain CD11b + inflammatory cells^13^. The *percent of neurons with neurites* (Fig. 5a), the *average neurite length* per neuron (Fig. 5b), and the *total neurite length* per field (Fig. 5c) were quantified from images of the immunolabeled cultures (3–4 wells in each of 3–4 biological replicates, ~1000 neurons/condition). Given the effect on neuron number observed with whole C3 plus myelin in rat early postnatal cortical cultures, the number of DRG neurons (β-TubulinIII+, Fig. 5d) was also quantified in these analyses.

For all proteins and concentrations tested, only C3b at 1 μg/ml (5.7 nM), significantly reduced the *percentage of neurons with neurites* in comparison to vehicle control (Fig. 5a; 69 ± 7%, *p = 0.043). This reduction in neurite outgrowth was associated with reduced neuron number (Fig. 5d; 61 ± 7%, **p = 0.005), suggesting a link to either neurotoxicity or impaired neuronal adhesion. Besides treatment with C3b, only the highest concentration of whole C3 exhibited a parallel reduction in neuron number (Fig. 5d; 64 ± 5%, **p = 0.001); this result may reflect the small amount of C3b produced by low-level spontaneous hydrolysis of C3 (Fig. 4). These data are the first demonstration of two novel roles for complement activation product C3b on neurons.

While C3 plus myelin treatment of rat early postnatal cortical neurons reduced *average neurite length* per neuron (Fig. 3b), treatment of adult mouse DRG neurons with C3b alone at the tested concentrations resulted in a trend in the expected direction that did not reach statistical significance (Fig. 5b; 100 ± 6%, 91 ± 6%, 87 ± 6%; p = 0.999, p = 0.216, p = 0.058, respectively). This difference would be consistent with a combinatorial effect of C3b and myelin as an inhibitory substrate. C3b reduced the *total neurite length* per field (Fig. 5c; 66 ± 6%, 54 ± 9%; *p = 0.027, **p = 0.005, respectively) at the same concentration effective for neurite initiation, however this output measure is directly sensitive to changes in neuron number. Surprisingly, treatment of DRG neurons with C3a resulted in an increase in *average neurite length* per neuron (Fig. 5b; 132 ± 6%, **p = 0.006), suggesting an opposing effect of this cleavage product in vitro. Critically, however, this effect reached significance at the middle dose only, at a 100-fold higher molar concentration than required for the C3b effect on *percentage of neurons with neurites*, and there was no parallel effect for the C3a rapid degradation product C3a desArg.

Taken together with the myelin-mediated cleavage of C3, these data suggest that C3b may be generated by proteases in the SCI microenvironment, and is sufficient to reduce both axon growth and viability/adherence of cultured neurons.

## Discussion

Our data suggest that complement protein C3 negatively regulates axon growth and neuron number both *in vivo* and *in vitro*. We present novel data regarding axon regeneration following SCI in C3 deficient mice, which display enhanced sensory fiber regrowth alongside potential neuroprotection. In culture, we demonstrate for the first time that C3 exacerbates myelin-mediated neurite outgrowth inhibition and adherent neuron loss, that C3 is cleaved by myelin-associated serine proteases, and that C3b alone is sufficient for neurite outgrowth inhibition and adherent neuron loss.

### Comparison with other relevant findings for complement in CNS injury

We have previously reported that complement C1q produces a separate, distinct effect on neurite outgrowth and axon regeneration. Whereas C3 directly reduced axon growth in vitro and following SCI, C1q reversed MAG-mediated outgrowth inhibition and repulsive growth cone turning in culture, and affected the guidance of regenerating axons *in vivo* ^13^. Importantly, C1q and C3 appear to affect axon growth through separate mechanisms that result in opposing effects. Although C3 activation is downstream of C1q in the well-described classical pathway of inflammatory complement activation, there is some evidence to suggest that C3 can also be regulated separately from other complement proteins in the spinal cord, including C1q^45^. Accordingly, the distinct effects observed may highlight the role of separable, specific, non-traditional functions for individual complement proteins after SCI^22^.

Histological and behavioral benefits of complement C3 deficiency on SCI recovery have been reported elsewhere, but importantly these studies did not assess axon dynamics including degeneration, regeneration, or sprouting^46^. The role of C3 in the axonal response to SCI has been briefly addressed in one publication^47^. In this study, C3 was reported to activate astrocytes, induce inflammatory signaling (TNF-α), and decrease neurite outgrowth *in vitro*, which were suggested to hinder locomotor recovery and regeneration in vivo; however, axon regeneration in vivo was not specifically quantified. Of interest, C3 signaling through CR3 has been associated with phagocytic synapse removal in the developing dLGN^48^ and in the post-injury ventral horn^45,49^, and C3 deficiency (but not C1q deficiency) was reported to reduce synaptic stripping of spinal motoneurons, increase GAP-43 expression, and improve functional recovery following nerve injury^50^, suggesting a non-traditional role for C3 in the spinal cord that could be predicted to affect both behavioral recovery and axonal connectivity. In summary, previous reports indicate detrimental effects for C3 on functional recovery and lesion dynamics after SCI, which is supported by our novel axon regeneration findings.

### Possible mechanisms for C3-mediated inhibition of axon growth

We report that treatment of neurons with purified C3b alone resulted in a reduction in *total neurite length* (Fig. 5c) and percentage of *neurons with neurites* (Fig. 5a), but did not affect *average neurite length* (Fig. 5b) unless it was combined with a myelin substrate (Fig. 3b). While *total neurite length* is dependent on the number of neurons present, percentage of *neurons with neurites* and *average neurite length* are not, because the data is expressed per neuron. These results are consistent with the known interactions of C3b with myelin proteins, suggesting a combinatorial effect of C3b and myelin as an inhibitory substrate. However, growth analyses that mathematically correct for the number of neurons present could still be linked to cell survival due to poor neuron health and energy metabolism, presence of cellular debris, alterations in neurotrophin signaling, apoptotic cascade activation, reduced electrical activity, and/or deficits in substrate adhesion^51,52^. Accordingly, because reduced neuron number was observed after culture of primary neurons on both myelin substrate plus whole C3 (Fig. 3c) and with purified C3/C3b (Fig. 5d), the possibility that neuronal cell death and/or complement-induced deficits in cell adhesion, via direct interference with substrate binding or indirectly following receptor mediated signal cascade activation, contributed to the observed outgrowth inhibition effects under these conditions cannot be excluded.

The traditional role for C3/C3b in cell death involves autocatalytic assembly of lytic C5b-9 MAC^35,53^. A second described role for complement in cell death involves anaphylatoxin (C5a, C3a, C4a) signaling through the G protein coupled transmembrane anaphylatoxin receptors (C3aR, C5aR) to either induce apoptosis directly, or to promote the release of inflammatory mediators (e.g. TNF-α, NOS) which injure adjacent cells^54–58^. Finally, a phagocytic mechanism for cell death via the opsonin functions of C3b in combination with CR3 expressing phagocytic cells is possible^35,59,60^. We were unable to detect C5b-9 (MAC, data not shown), CR3/CD11b+ inflammatory cells^13^, or complement receptors known to stimulate phagocytosis (CR1/CR2, CR3, and C3b/iC3b; data not shown), and observed less than 0.5% Iba-1+ macrophages/microglia^13^ in our primary cultures, making direct lysis and phagocytic removal unlikely mechanisms of cell death in this paradigm. These data are consistent with the study design, as all *in vitro* experiments used primary neuronal cultures that were serum free. Additionally, our data do not support cell death via an anaphylatoxin signaling mechanism because although C3aR is expressed by neurons^54,61,62^, C3a was not toxic at the concentrations tested, and importantly, C3a actually increased neurite outgrowth in vitro. A role for direct phagocytosis cannot be completely excluded, as both astrocytes and Schwann cells have been demonstrated to possess phagocytic capacity^63–67^, and are likely to be present in our DRG cultures based on the presence of p75+/βTubulinIII- and S100+/βTubulinIII- immunolabeled cells (data not shown), as well as previous reports^66^. Finally, rather than promote direct toxicity, it is possible that C3/C3b instead causes deficits in cell adhesion, either by direct interference or indirectly following receptor mediated signal cascade activation.

In terms of C3/C3b toxicity *in vivo*, the failure to detect significant DRG neuron loss in genetically normal animals following sciatic nerve injury (Fig. 2) is in contrast to some reports from the literature. This discrepancy may reflect insufficient power to detect a small difference between injured and uninjured DRGs in our C3^+/+^ group. In addition, species and strain differences could account for the discrepancy between the present study and previous reports, as the majority of studies characterizing DRG neuron loss after sciatic nerve injuries have been performed in rats^30–34,68^, and no mouse sciatic nerve injury experiments have used the BUB/BnJ strain, which is more complement sufficient than other mouse strains^69,70^. Further, neuroprotective phenotypes have been reported for C3 deficient mice using other injury/disease models^71,72^. Accordingly, we cannot exclude the possibility that C3 deficiency could have neuroprotective effects after injury, as such an observation would be consistent with our *in vitro* results. The overall importance of cellular survival on the neurite growth inhibitory function of C3 therefore requires further investigation.

### C3 cleavage by myelin and the effect of cleavage proteins on axon growth at physiological concentrations

Traditionally, C3 is cleaved by the C3 convertase, a serine protease produced by the formation of a complex of either C4b + C2b (classical and lectin pathways) or C3b + Bb (alternative pathway). However, other cationic, neutral and serine proteases have been suggested to substitute for C3 convertase activity, and serine proteases such as neurosin and myelencephalon-specific protease have been identified in myelin^38–44^. Accordingly, we report the first direct demonstration of C3 cleavage by myelin-associated serine protease activity, and demonstrate the potential for disrupted myelin following SCI to potentially enhance activation of complement proteins, promoting diverse inflammatory and non-traditional functions, and affecting histological and behavioral recovery.

Interestingly, while C3b reduced the percentage of *neurons with neurites* in our DRG cultures, C3a increased the *average neurite length* per neuron (Fig. 5). Although this novel C3a result is surprising, a recent paper has suggested that C3a and C3aR positively regulate axon growth-associated protein GAP43 as well as synapsin^+^ and VGLUT1^+^ synaptic density following experimental photothrombotic stroke in mice^73^. In addition, C3a has been shown to stimulate nerve growth factor (NGF) expression in cultured microglia^74^ and astrocytes^75^, which could improve neuroprotection in the injured or diseased CNS^74,75^; importantly, NGF can also enhance sensory axon regrowth, and could thus be a potential mechanism for C3a-mediated neurite growth in our sensory neuron cultures. It is intriguing that two proteins simultaneously generated from C3 cleavage exhibited opposing effects on neurite growth, and that while the C3b inhibitory effect was associated with changes in neuron number, the C3a growth effect was not. Complement anaphylatoxins have been implicated in cytoskeletal rearrangement, integrin production, and neurotrophin synthesis, which may underlay neurite growth^54^. The distinction between the analysis of *average neurite length* versus percent of *neurons with neurites* is potentially informative, as C3b and C3a each affected one measure of neurite growth but not the other. Complement C3b may negatively affect the initiation of neurite growth from the soma, while C3a could positively impact the rate of axon elongation (versus degeneration) through distinct mechanisms^76,77^. As described below, the effective molar concentration of C3 and C3b tested was significantly lower than that of C3a and C3a desArg, meaning that approximately 100x fewer C3b molecules were required to affect axon outgrowth in comparison to C3a. In addition, C3a is small, soluble, diffuses away from C3 cleavage and rapidly inactivates (forming C3a desArg) *in vivo*, but C3b is relatively large and binds to cell membranes^35,59^. Thus, specific effects on axon growth in response to individual C3 products may be predicted to exist based on concentration, location and timing *in vivo*. It is important to note that the other experiments reported in this manuscript, e.g. the addition of whole C3 to cortical cultures with myelin and the genetic absence of C3 after SCI, generated data consistent with the growth inhibitory effect for C3, which appears to be mediated by C3b and not C3a. Therefore, when combined, the C3b effect may be dominant over the C3a effect, or the assays and analyses used were either not sensitive enough or not appropriate for detecting the C3a effect.

The concentration range of whole C3 protein and proteolytic fragments exogenously applied to our primary neuronal cultures was chosen based on estimates from physiological availability and culture dynamics. First, complement C3 circulates in human serum at 1 mg/ml (2.72 μM)^78^, and is secreted by inflammatory cells and resident CNS cells at presumably lower, but not well-described concentrations following injury^22^. Although it is unclear from the literature the exact extent of neuronal exposure to complement at various distances from the lesion after SCI due to the combination of blood spinal barrier (BSB) disruption, inflammatory infiltration, and local synthesis, the neurite outgrowth data reported here encompass a range of 0.01–250 μg/ml (54pM-680 nM) C3, which is physiologically plausible *in vivo*. A single C3 molecule produces a single C3a fragment and a single C3b fragment upon cleavage, and subsequently, a single C3a desArg fragment is produced from C3a cleavage. C3b fragments were used at 0.01–1.0 μg/ml (57 pM-5.7 nM), C3a fragments were used at 0.01–1.0 μg/ml (1–111 nM), and C3a desArg fragments were used at 0.01–0.1 μg/ml (1–11 nM). Therefore, although the concentrations of these molecules administered were the same, the molar concentration of C3 and C3b tested was much lower than that of C3a and C3a desArg. In summary, all C3 and C3 fragment concentrations tested in our culture experiments were less than that for C3 in human serum, and are estimated to be physiologically relevant following SCI trauma.

We conclude that C3 and other complement proteins are likely participate in non-traditional roles as mediators of axon regeneration and neuronal survival/adhesion, adding to other recently described non-immune functions for complement. Additionally, we report the novel finding that serine proteases present in myelin and myelin debris are capable of cleaving C3 to generate activation products, suggesting a new mechanism by which both complement cascade activation and more targeted complement molecule cleavage product activities may be initiated in the CNS. Future studies testing motor tract axon regeneration, locomotor recovery and neuronal survival after SCI using specific pharmacological inhibition of C3b will be crucial in determining the therapeutic impact of these findings.

## Electronic supplementary material

Supplemental Materials and Methods
