# Supplementary material for: Complement Protein C3 Suppresses Axon Growth and Promotes Neuron Loss
Source: Sci Rep. 2017 Oct 10;7:12904. doi: 10.1038/s41598-017-11410-x (PMC5635131; doi:10.1038/s41598-017-11410-x)
Supplement: Supplementary file 1 — Supplemental Materials and Methods [file 41598_2017_11410_MOESM1_ESM.pdf]

Title: Complement Protein C3 Suppresses Axon Growth and Promotes Neuron Loss.

Author Names: Sheri L. Peterson, Hal X. Nguyen, Oscar A. Mendez, Aileen J. Anderson*

SUPPLEMENTAL INFORMATION

SUPPLEMENTAL MATERIALS AND METHODS

Dorsal hemisection SCI with sciatic nerve conditioning injury

Male C3 deficient mice[28] were purchased from Jackson Labs and backcrossed using a Max-Bax strategy onto the BUB/BnJ background at Charles River Laboratories (San Diego) until congeneic. The BUB/BnJ strain was chosen because total hemolytic complement activity in males of this strain is closest to those in rats and humans, while most common mouse strains exhibit much lower levels[29,30]; in this regard, we have previously reported that while male BUB/BnJ mice exhibit hemolytic complement activity in response to SCI, female mice and male C57BL/6 mice do not. C3 deficient BUB/BnJ mice exhibit a complete absence of C3 protein after SCI, although C5b-9 deposition is detectible (Anderson, unpublished observations).

Axon regeneration immunohistochemistry & analyses

Immunohistochemistry: For analyses of regenerated axons, a series of every 2nd section of the T4-T9 spinal cord segments (11-16 sections per mouse, 50 μm apart) was processed for CTB immunoreactivity, with the starting section number chosen for each mouse at random. Individual free-floating sections were washed, endogenous peroxidases inactivated (3%H202/10%Methanol), permeabilized (TritonX100), blocked (BSA and donkey serum), and incubated in goat anti- cholera toxin β subunit (List Biological Laboratories, 1:8000) for 15 h; followed by 1 h incubation with each biotinylated donkey anti-goat IgG $F(ab')_2$ fragment (Jackson ImmunoResearch, 1:500), then avidin-biotin complex (Vectastain), before visualization using 3,3'-Diaminobenzidine enhanced with nickel chloride. Every 6th section through the medulla (7-12 sections per mouse, 150 μm apart) was immunolabeled using this procedure, and any animals with incomplete dorsal column transection, as indicated by the presence of spared fibers in the gracile nucleus, were excluded as described below. In addition, every 3rd section of the lumbar spinal cord (6-9 sections per mouse, 100 μm apart) was processed for CTB immunoreactivity. From these sections, mice with absent or insufficient tracer labeling were excluded as described below, and tracer uptake/transport in $C3^{+/+}$ versus $C3^{-/-}$ mice was compared semi-quantitatively. Negative tissue controls as well as omission of primary antibody were used to confirm the specificity of the primary antibody and secondary antibodies in our protocol. *Microscopic Analyses*: Histological sections were examined on an Olympus BX60 microscope. Length of the longest CTB^{+} axon was determined by visually scanning each spinal cord section (200x-400x magnification; full 1:2 section sampling; MicroBrightField v9.2 Software), and measuring rostro-caudal length from the center of the injury to the most rostral axonal tracer label in each animal. The center of the injury was set to 0 μm, with rostral positions reported as positive and caudal as negative. Lesion volume was determined using the unbiased, systematic random sampling methods of stereology with the Cavalieri probe (MicroBrightField v9.2 Software; 40x-100x magnification; 1:2 section sampling through entire lesion). Using a 100 μm grid, Gunderson CE m=1 ranged 0.015 - 0.071 for all mice. To exclude mice with incomplete dorsal column lesions, medulla sections were checked for evidence of tracer by manual scanning (100x-400x magnification), and any mouse with CTB immunoreactivity was excluded (N=7 excluded mice: 3 $C3^{-/-}$, 2 $C3^{+/+}$, and 2 $C3^{+/+}$ SCI only). To exclude mice with poor tracer injections or transport, lumbar sections were manually scanned (100x-400x magnification) for tracer immunoreactivity, and all mice without distinct, intense black axon labeling were excluded from all analyses (N=2 excluded mice: 1 $C3^{-/-}$, 1 $C3^{+/+}$ SCI only). Additionally, gross analysis of the lumbar tracer label between genotypes was performed two ways: by semi-quantitative rating of the amount of label on the most highly stained section per mouse, and also by calculating the percent of all lumbar sections containing any positive tracer immunolabel. N=4 $C3^{+/+}$ mice, including 1 with SCI only, either died during surgery or were euthanized due to bladder complications during the course of the study, and tissues from these mice were not processed for analysis.

Sciatic nerve transection, immunohistochemistry, and analysis of DRG survival

For estimates of total DRG neuron number, a series of every 4th section of each DRG (4-11 sections / DRG, 90 µm apart) was processed for NF200 immunoreactivity, with the starting section number chosen for each mouse at random. Slide-mounted sections were pre-treated with Buffer A (Electron Microscopy Sciences) to remove tape and retrieve antigens. Slides were then washed, permeabilized (TritonX100), blocked (BSA and donkey serum), and incubated in rabbit anti-neurofilament 200 (Millipore, 1:500) for 18 h; followed by 1 h incubation with AlexaFluor 488 conjugated donkey anti-rabbit IgG F(ab')$_2$ fragment (Jackson ImmunoReasearch, 1:500) plus Hoechst 33342 (10 µg/mL), before final wash and coverslip with Fluoromount-G. Omission of primary or secondary antibody was used to confirm the specificity of the primary and secondary antibody in our protocol.

Isolation of myelin substrate

Myelin was isolated and purified from the cerebellum of an adult female Sprague Dawley rat brain by sucrose gradient (1.9 M, 1.4 M homogenate layer, 0.85 M, 0.25 M) separation of the tissue homogenate (14 h 40,000 rpm 4°C in gradient, followed by 1 h 40,000 rpm 4°C in dH$_2$0) in the presence of protease inhibitors (Calbiochem), according to a previous study[32]. Myelin protein concentration was determined by BCA protein assay (Pierce), and varying concentrations were tested for growth inhibition effects, and the 7.5 µg/mL (1.12 µg/well) concentration was chosen for reported experiments.

Immunocytochemistry & quantification for in vitro neurite analyses

Fixed cell culture wells were washed with PBS, and PBS washes were performed between each step described. After blocking (30 m; 3% BSA, 0.05% Tween-20), cells (DRG or cortical) were immunolabeled using rabbit anti-β-tubulin class III (Covance; 1:1000 in buffer with detergent, BSA and gelatin; 1 h) primary antibody to label a neuronal microtubule protein, followed by anti-rabbit IgG conjugated to Alexa-488 (Invitrogen; 1:1000; 1 h) secondary antibody for visualization. Hoechst 33342 (10 µg/mL; 30 min) was used as a nuclear stain. After final PBS washes, slides were coverslipped using Fluoromount G (SouthernBiotech). Images (100x or 200x magnification) of Hoechst and β-tubulinIII were captured on either an Olympus IX71 (8 pre-determined spots / well) or a Zeiss Axio Imager.M2 microscope (9 auto-captured, pre-determined sports / well), and used to quantify cell number and neurite length. Counts for number of β-tubulinIII+ and Hoechst+ cells were determined either by hand or using a custom protocol in Bitplane Imaris (v7.3.1 software) and visually checked for errors. *Longest neurite length per neuron* was determined using ImageJ software, by individually measuring the longest neurite for each β-TubulinIII+ cell with growth. *Neurite count per neuron* reflects the average number of neurites per neuron with growth (starting from the cell body and longer than the diameter of the soma), and was determined either by hand or using a custom protocol in Bitplane Imaris. *Total neurite length per well* was determined using ImageJ with the Neurite Tracer plug-in or using a custom protocol in Bitplane Imaris. *Average neurite length per neuron* was calculated by dividing total neurite length by #β-tubulinIII+ cells for each well.
